# Development of anoikis-related long non-coding RNA signature associated with prognosis and immune landscape in cutaneous melanoma patients

**DOI:** 10.18632/aging.204932

**Published:** 2023-08-04

**Authors:** Like Zhong, Wenkang Qian, Wangang Gong, Li Zhu, Junfeng Zhu

**Affiliations:** 1 Zhejiang Cancer Hospital, Hangzhou Institute of Medicine (HIM), Chinese Academy of Sciences, Hangzhou 310022, Zhejiang, China

**Keywords:** cutaneous melanoma, anoikis-related lncRNAs, risk model, prognosis, immune landscape

## Abstract

Background: Anoikis is involved in many critical biological processes in tumors; however, function in CM is still unknown. In this study, the relevance between Anoikis-related lncRNAs (ARLs) and the clinicopathological characteristics of patients with CM was comprehensively assessed.

Methods: Through analysis of TCGA dataset, ARLs were identified by using TCGA dataset. Based on the ARLs, a risk model was established to anticipate the prognosis of patients with CM, besides, the prediction accuracy of the model was evaluated. The immune infiltration landscape of patients with CM was assessed comprehensively, and the correlation between ARLs and immunity was elucidated. Immunotherapy and drug sensitivity analyses were applied to analyze the treatment response in patients with CM with diverse risk scores. Different subgroups were distinguished among the patients using consensus cluster analysis.

Results: A risk model based on six ARLs was set up to obtain an accurate prediction of the prognosis of patients with CM. There were distinctions in the immune landscape among CM patients with diverse risk scores and subgroups. Six prognosis-related ARLs were highly correlated with the number of immune cells. Patients with CM with different risk scores have various sensitivities to immunotherapy and antitumor drug treatments.

Conclusion: Our newly risk model associated with ARLs has considerable prognostic value for patients with CM. Not only has the risk model high prediction accuracy but it also indicates the immune status of CM patients, which will provide a new direction for the individualized therapy of patients with CM.

## INTRODUCTION

Cutaneous melanoma (CM) is a malignant tumor originating from melanocytes and is the third most common skin malignancy after basal cell carcinoma and squamous cell carcinoma [1–3]. In 2020, over 320,000 people were diagnosed with CM, and the fatalities are nearly 60,000 [4]. CM has become a major problem endangering human health [4]. The highly invasive and metastatic nature of CM is responsible for the 5-year overall survival (OS) of only 23% of CM patients [5]. As the most aggressive malignant tumor, CM has obvious heterogeneity [1, 6]. Invasion and metastasis are associated with high mortality in CM owing to the lack of effective treatments [7]. Targeted therapy and immunotherapy have achieved remarkable results in treating CM; however, these therapeutic methods cannot solve the problem of tumor invasion and metastasis [8]. Notably, CM has high late mortality and early cure rates, therefore the early detection, diagnosis, and treatment of CM will have great clinical significance [9, 10].

It is well known that the mortality rate of tumors is the highest among all diseases and local recurrence or metastasis of tumors was the main reason for the poor prognosis of patients with malignant tumors [11, 12]. During invasion and metastasis, tumor cells must overcome many difficulties, especially death caused by detachment from the extracellular matrix (ECM), also known as anoikis [13, 14]. Anoikis is a special type of programmed apoptosis initiated by cells detaching from the ECM and has significant influence in the development of the body, tissue homeostasis, disease occurrence, and tumor metastasis [15, 16]. Anoikis is indispensable for maintaining tissue homeostasis, and its main role is to hinder abnormal cell growth or adhesion to the abnormal extracellular matrix [15]. Normal cells are adhesion-dependent, and their survival depends on anchorage-dependent signaling between cells and between cells and the matrix [17]. When normal cells or solid tumor cells without metastatic properties are shed from situ into the blood, apoptosis will be triggered, while malignant cells can escape anoikis [18, 19]. The mechanism of anoikis resistance in malignant tumor cells have elucidated by abundant studies, especially in anoikis-resistant tumor cells that exhibit stronger invasive and metastatic abilities [20, 21]. Therefore, studies on anoikis will contribute to the effective treatment of malignant human tumors. CM has always plagued us due to its local recurrence and metastasis [9, 10]. Tumor invasion and metastasis in CM and the impact of anoikis on the prognosis of CM are key to CM treatment; the clinical value of anoikis in CM has not received extensive attention, however.

Long non-coding RNAs (lncRNAs are functional RNAs over 200 nucleotides in length, and have strong transcriptional control capabilities during development and gene expression [22–24]. Particularly, it plays a complex and precise regulatory function in tumorigenesis and progression [15, 25, 26]. Recently, increasing evidence has suggested that lncRNAs can rule out disease heterogeneity and act as markers for many tumors [27, 28]. As more lncRNAs have been characterized, it is obvious that they play important roles in the tumor invasion-metastasis cascade, distant organ colonization, and TME [29]. LncRNAs can exert their effects on cancer pathways through different mechanisms, but unanswered questions remain, such as the role of lncRNAs in anoikis [29]. Notably, currently no effective risk prediction model is confirmed for patients with CM based on anoikis that can comprehensively reflect the effect of anoikis-related lncRNAs (ARLs) on prognosis. Therefore, it is important to identify reliable biomarkers using ARLs to speculate the prognosis and progression of patients with CM.

A number of reports have pointed out the influence of anoikis-related genes or lncRNAs on immune infiltrating microenvironment [30, 31]. However, the mechanism has not been reported in depth. The interaction between tumor cells and their microenvironment is important for cell survival [32]. LncRNAs are important players in the crosstalk between cancer cells and the tumor microenvironment (TME). By promoting the formation of immunosuppressive tumor immune microenvironment (TIME), lncRNAs contribute to tumor escape immune surveillance, thereby influencing subsequent tumor metastasis, development, and drug resistance to treatment [33, 34]. LncRNAs affect the development and function of immune components including macrophages, T cells, and cancer-associated fibroblasts (CAF), which leads to the possibility to affect anoikis resistance through the regulation of immune components [30].

According to this study, the relationship between ARLs and the clinicopathological characteristics of patients with CM was comprehensively assessed through the analysis of CM data which was obtained from The Cancer Genome Atlas (TCGA) database. Based on these 6 ARLs, we established a novel risk model and validated its capability to anticipate OS in patients. Additionally, we assessed the immune landscape of patients through multiple algorithms to further explore the relevance and importance of anoikis in tumor immunity. Immunotherapy and drug sensitivity analyses provided a reference for the treatment of patients with CM. In conclusion, new clinical insights into anoikis in CM and a new path for the treatment of CM are suggested by the results of this study provide suggest.

## MATERIALS AND METHODS

### Data collection and processing

We followed the methods of Ma et al. [35]. The transcriptome data (TPM) as well as clinical information of 454 patients involved in this study was collected from The Cancer Genome Atlas (TCGA, https://portal.gdc.cancer.gov/). 34 anoikis-related genes (ARGs) were downloaded from the molecular signature database (https://www.gsea-msigdb.org/gsea/). LncRNAs were differentiated from expression profiles using Perl scripts for subsequent screening and matched with relevant clinical information.

### Construction and validation of prognostic risk model based on ARLs

To screen for prognosis-related ARLs, we analyzed the relationships between these 34 ARGs and all lncRNAs. Univariate Cox regression as well as least absolute shrinkage and selection operator (LASSO) analyses was used to evaluate the prognostic value of ARLs (*p*<0.05), which indicated that lncRNAs had significant connection with CM prognosis. Multivariate Cox regression was applied to screen for ARLs with independent prognostic value and to construct risk models. The sample risk score formula was as follows: risk score = ∑_i_=Coef_i_ ∑ (expression of lncRNA_i_). Patients with CM were distributed into low- and high-risk groups on the basis of the median risk scores. Kaplan-Meier survival curves were analyzed and visualized applying the–plan “Meier survivalROC” package. Principal component analysis (PCA) was used to analyze the segregation patterns in CM patients with various risk scores. To obtain a further verity of the prediction accuracy of the risk model, the samples were distributed into training and test cohorts at a 7:3 ratio, and the samples of each group were re-divided into high- and low risk groups based on the median value of the risk score in each group [36].

### Construction of nomogram based on prognosis-related ARLs

Univariate and multivariate Cox regression analyses were applied to evaluate the independent prognostic value of risk scores or related clinical information, including age, sex, and stage. On the basis of independent prognostic factor, the nomogram was established by the “rms” package. The concordance index (C-index) and calibration curve were applied to assess nomogram accuracy. The prognostic prediction ability of the risk model was assessed by the “timeROC” package.

### Enrichment analysis

Gene Set Variation Analysis (GSVA) was used to assess the enrichment of different signaling pathways across the samples. Kyoto Encyclopedia of Genes and Genomes (KEGG) was used to identify potential signaling pathways. Gene Ontology (GO) can help us have a further understanding of the biological effects of genes.

### Consensus clustering

The Consensus Clustering method is widely used for tumor analysis. Cluster analysis was performed on the resampled samples by the “ConsensusClusterPlus” package, and CM patients were divided into different subtypes for further analysis according to the clustering results of K=2 to 9.

### Immune landscape analysis

CIBERSORT and ESTTIMER were used to speculate the immune infiltration landscape of patients with CM. Immune scores as well as tumor purity of CM patients were analyzed by the “estimate” package. The abundances of 22 immune cells were assessed using the CIBERSORT algorithm. The proportions of 23 immune cells were evaluated by the Single-Sample Gene Set Enrichment Analysis (ssGSEA) algorithm.

### Immunotherapy and drug sensitivity analysis

Immunotherapy results were collected from the TCIA database (https://tcia.at/home). The “limma” package is used to extract expression data of relevant immune checkpoint inhibitors (ICIs) from the matrix. Drug sensitivity analysis was used to speculate the therapeutic response of patients with CM to different drugs and was quantified using the half-maximal inhibitory concentration index (IC50).

### Statistical analysis

All statistical analyses pertain to this study were performed by using R software v4.1.2. We used the Pearson correlation method to estimate the correlation of anoikis and lncRNAs. The correlation between ARLs and immune cell fraction was evaluated utilizing Pearson analysis. The Wilcoxon rank-sum test was applied to assess the differences between the two groups, and *p*<0.05 would be regarded statistically significant.

### Data availability

The datasets used in this study were obtained from a public database (The TCGA, https://portal.gdc.cancer.gov/). The original contributions presented in this study are included in the article or Supplementary Material. Further inquiries can be directed at the corresponding author.

## RESULTS

### Identification of prognostic ARLs

**Figure 1:**
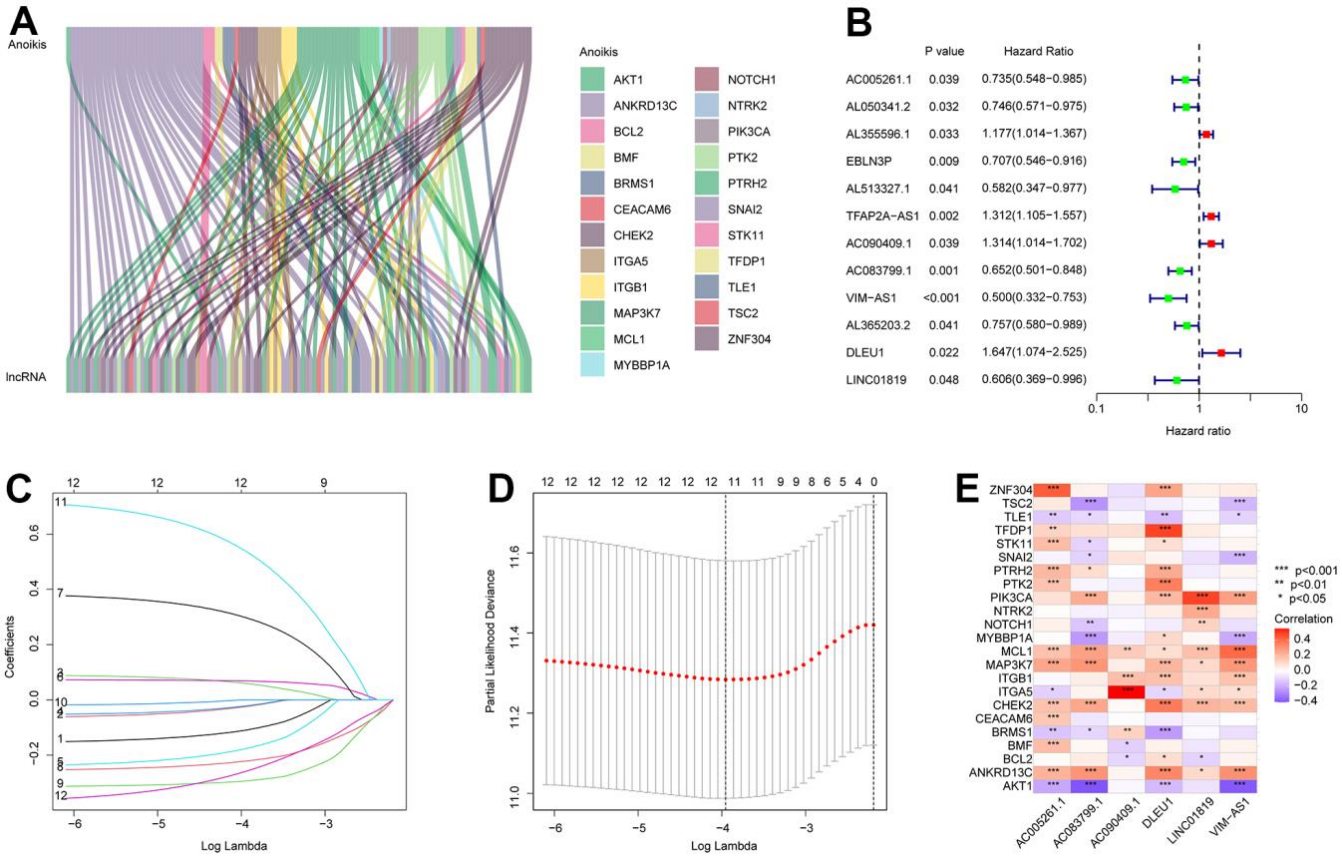
**Identification of prognostic ARLs in CM.** (**A**) Identification of ARLs in CM. (**B**) Univariate Cox regression analysis shows that 12 ARLs are associated with OS rate. (**C**, **D**) LASSO shows the optimal coefficients and minimum lambda of the prognostic ARLs. (**E**) Correlation analysis of the ARLs and anoikis genes.

In this study, 14,142 lncRNAs were obtained from the RNA-Seq matrix of CM. To identify the lncRNAs associated with anoikis, the correlation between 34 anoikis genes and lncRNAs was calculated, and 88 lncRNAs were identified as ARLs (Figure 1A). Based on the univariate Cox regression analysis, 12 ARLs which were associated with the OS rate were identified via least absolute shrinkage and selection operator (LASSO) analysis (Figure 1B–1D). Among these, six prognostic ARLs being able to independently anticipate the prognosis of patients with CM were selected using multivariate Cox regression analysis. Correlation analysis suggested that the six prognostic ARLs were closely related to anoikis genes (Figure 1E).

### Risk model construction of ARLs

**Figure 2:**
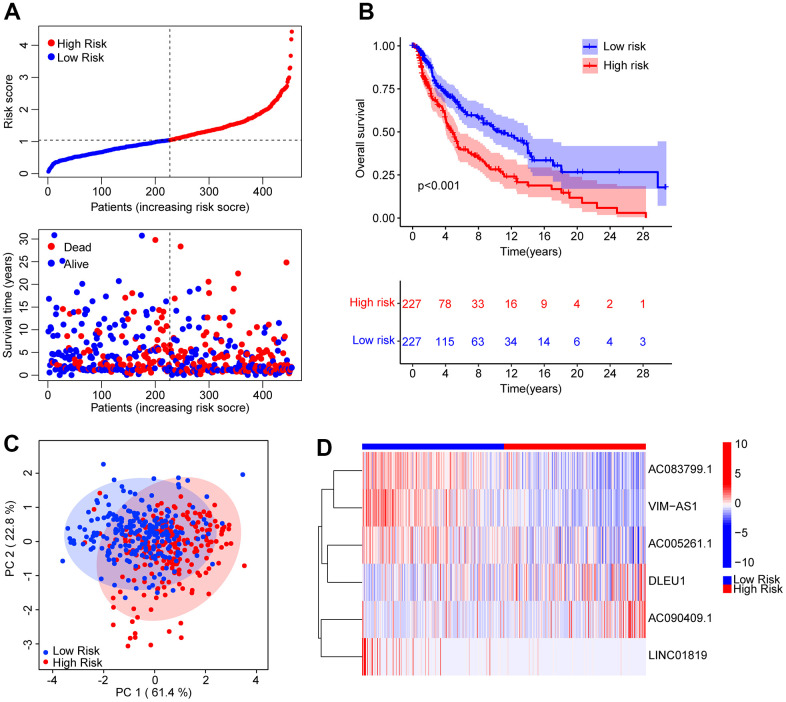
**Risk model construction based on the prognostic ARLs in CM.** (**A**) The distribution of the CM patients and the scatter dot plot shows the correlation between the risk score and survival time in CM. (**B**) The Kaplan-Meier survival curve shows that the OS rate of patients in the low-risk group is remarkably higher than those in the high-risk group. (**C**) It is illustrated that there is a clear distribution of patients in the low- and high-risk group based on the prognostic ARLs by principal component analysis (PCA). (**D**) The expression of the prognostic anoikis-related lnc7RNAs in the low- and high-risk group can be reflected by the heatmap diagram.

A novel risk model was founded to estimate ARL prognosis in patients with CM. On the basis of the median risk score, patients with CM were ranked and distributed into low- and high-risk groups. It was suggested by the scatter dot plot that the risk score was inversely correlated with survival time for CM (Figure 2A). Kaplan-Meier survival curve analysis elaborated that the OS rate of patients with a low-risk score was remarkably higher than that of patients with a high-risk score (Figure 2B). Principal component analysis (PCA) showed an obvious partition between the patients in the low- and high-risk groups based on prognostic ARLs (Figure 2C). Heatmap visualization revealed remarkable differences in the six prognostic ARLs between the low- and high-risk groups (Figure 2D). The low-risk groups exhibited higher AC083799.1, VIM−AS1, AC005261.1, and LINC01819 expressions, while the expression of DLEU1 and AC090409.1 was higher in the high-risk group. It is demonstrated that the risk model construction which is based on the prognostic signature of the six ARLs can precisely predict the prognosis of patients with CM.

### Risk model construction in the training cohort and validation cohort

**Figure 3:**
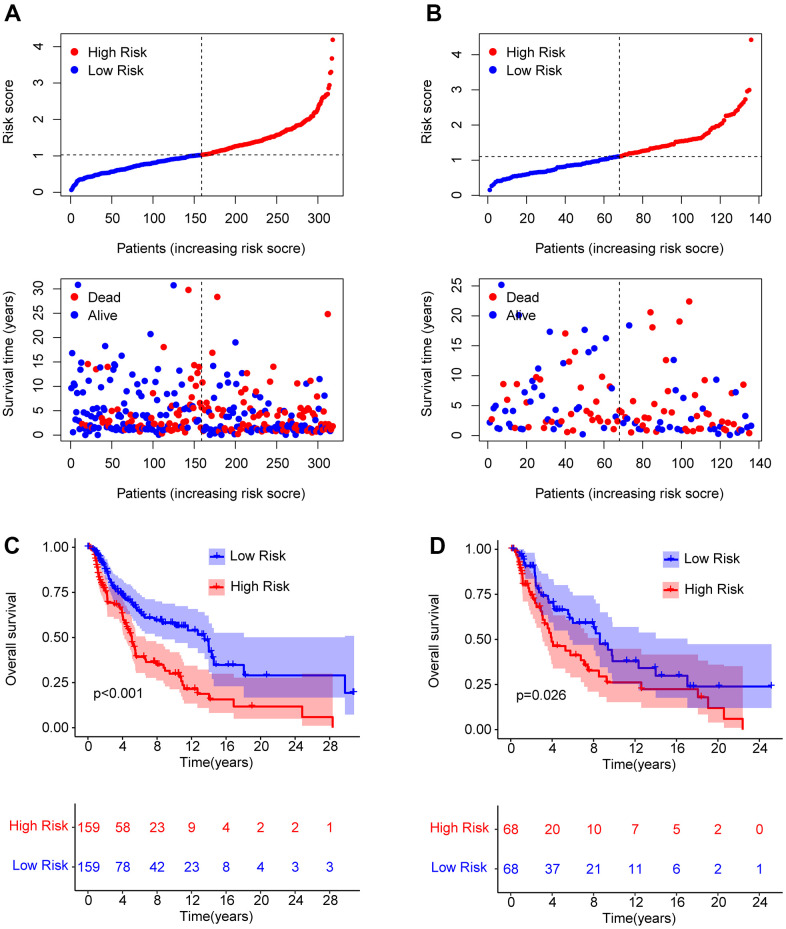
**Risk model construction in the training cohort and test cohort based on the prognostic ARLs.** The classification of the CM patients and the scatter dot plot demonstrates the correlation between the risk score and survival time of CM patients in training cohort (**A**) and test cohort (**B**). (**C**, **D**) The Kaplan-Meier survival curve shows the OS rate of patients in the training cohort and test cohort.

To obtain further evaluation of the precision as well as independence of the risk model for anticipating the prognosis of patients with CM on the basis of the ARLs, an internal validation model was used. Patients with CM were randomly distributed into training and validation cohorts, with 318 and 136 samples in the training and validation cohorts, respectively. In line with the prognostic signature of ARLs, patients with CM were ranked and classified into low- and high-risk groups in both cohorts. The scatter dot plot suggested that the risk score based on ARLs had an inverse correlated with the survival time of patients with CM in the training and validation cohorts (Figure 3A, 3B). Kaplan-Meier survival curve analysis revealed that the OS rate of patients with CM with low-risk scores was remarkably longer than that of patients with high-risk scores (Figure 3C, 3D). Given the results above it is demonstrated that the risk model based on the prognostic ARLs for evaluating the prognosis of patients with CM is accurate and reliable.

### The prognostic signature based on the ARLs was an independent prognostic indicator for CM

**Figure 4:**
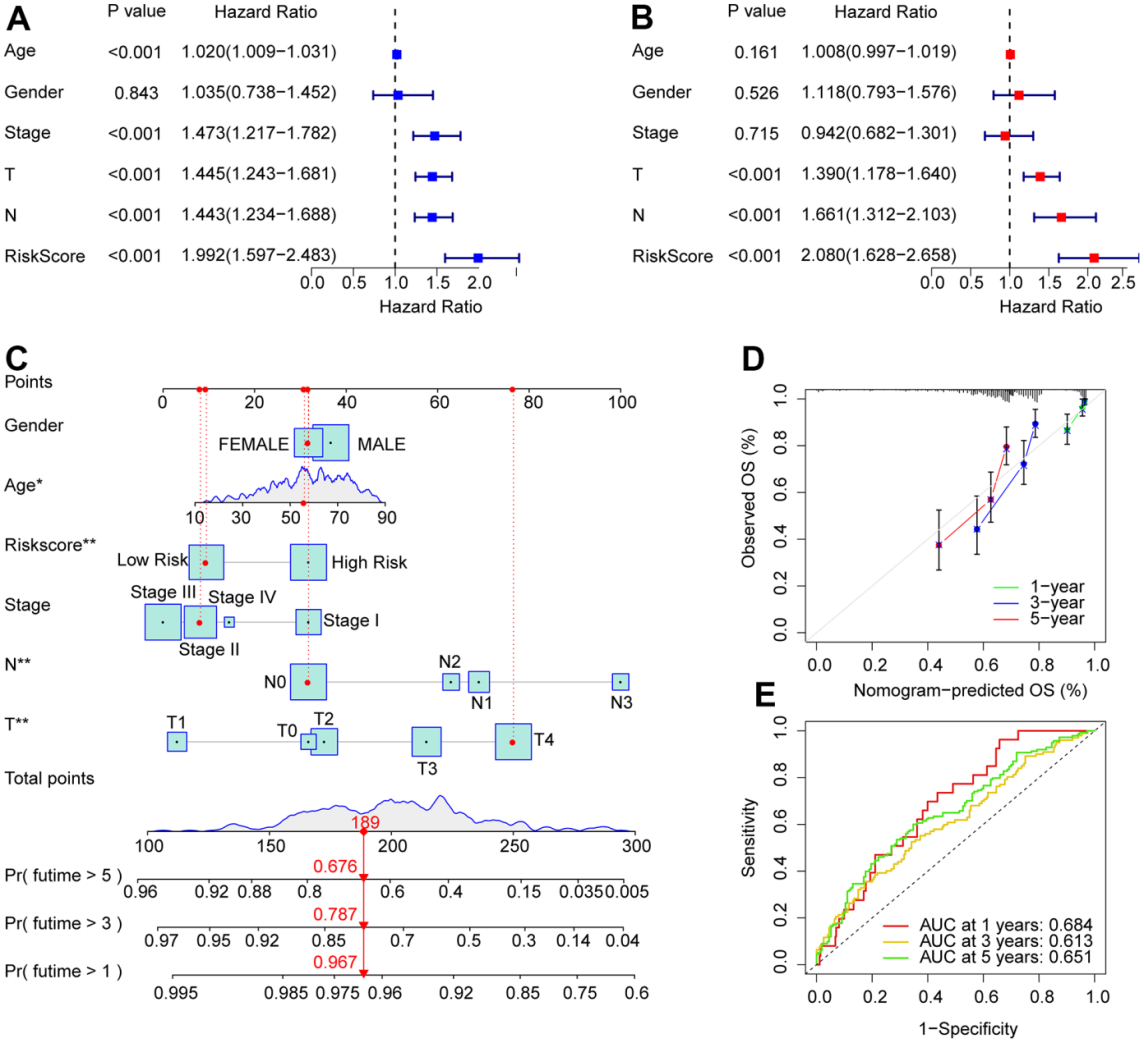
**Independent prognosis analysis of the ARLs prognostic signature and clinicopathological characteristics.** (**A**) Univariate Cox regression shows that age, stage, T, N, and risk score are connected with OS in CM. (**B**) Multivariate Cox regression suggests that T, N, and risk score are independent prognostic indicators for CM. (**C**) Nomogram model construction based on the different clinicopathological characteristics and ARLs prognostic signature. (**D**) The calibration curve shows the consistency between the predictive power and actual survival of 1, 3, and 5 years. (**E**) Time-dependent ROC curves show the AUC at 1, 3, and 5 years.

Univariate and multivariate Cox regression analyses were used to investigate the risk score as a reliable prognostic indicator of CM. Univariate Cox regression analysis showed that age (hazard ratio (HR) = 1.020, *P*< 0.001), stage (HR = 1.473, *P*< 0.001), T stage (HR = 1.445, *P*< 0.001), N stage (HR = 1.443, *P*< 0.001), and risk score (HR = 1.992, *P*< 0.001)had a close connection with the OS rate in CM (Figure 4A). Multivariate Cox regression analysis demonstrated that T (hazard ratio [HR] = 1.390, *P*< 0.001), N (HR = 1.661, *P*< 0.001), and risk score (HR = 2.080, *P*< 0.001) were independent prognostic predictors in CM patients (Figure 4B). Moreover, a nomogram was founded to precisely predict the 1-, 3-, and 5-year survival of patients with CMon the basis of the ARLs prognostic signature and clinicopathological characteristics (Figure 4C). Calibration curves showed satisfactory consistency between the 1-, 3-, and 5-year OS rates anticipated by the nomogram model and the accurate OS rate for patients with CM (Figure 4D). Time-dependent ROC curves suggested that the AUC at 1, 3, and 5 years were 0.684, 0.613, and 0.651, respectively (Figure 4E). Collectively, these results demonstrated that the risk score based on prognostic ARLs was independent as a prognostic indicator and could precisely estimate the survival time of patients with CM.

### Subgroup analysis of ARLs prognostic signature and clinicopathological characteristics

**Figure 5:**
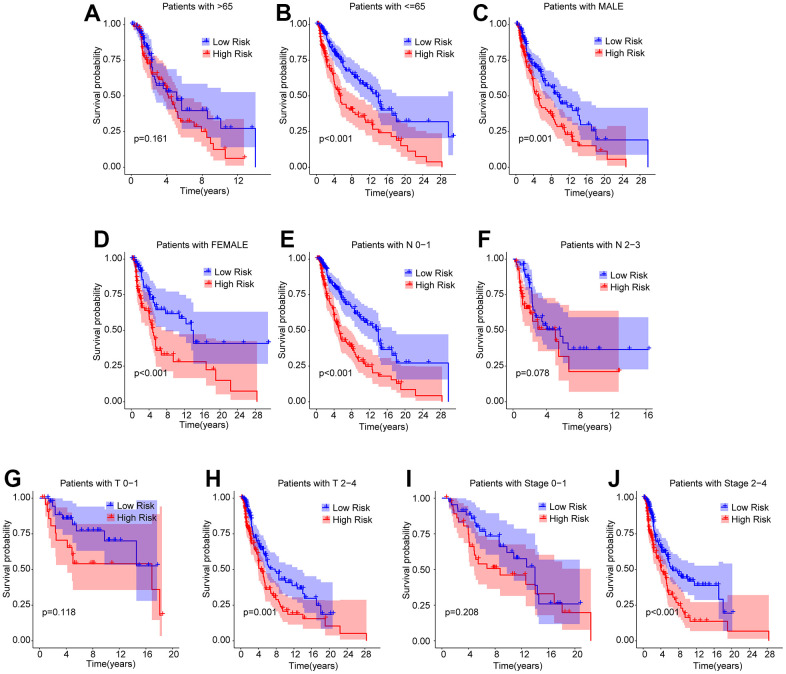
**Subgroup analysis of CM patients with low- and high-risk scores in different clinicopathological characteristics.** The Kaplan-Meier survival curve shows the OS rate of CM patients in the low- and high-risk group stratified by (**A**, **B**) Age (> 65 vs ≤ 65); (**C**, **D**) Gender (male vs female); (**E**, **F**) N (N 0–1 vs N 2–3); (**G**, **H**) T (T 0–1 vs T 2–4); (**I**, **J**) Stage (stage 0–1 vs stage 2–4).

A stratified subgroup analysis was conducted to investigate the independence of the risk score in evaluating the prognosis of CM patients with clinicopathological characteristics. Based on the ARLs prognostic signature, the CM patients were distributed into the two risk subgroups according to the different clinicopathological characteristics, including age (> 65 vs≤ 65), gender (male vs female), N (N 0–1 vs N 2–3), T (T 0–1 vs T 2–4), and stage (stage 0–1 vs stage 2–4). As shown in Figure 5, the Kaplan-Meier survival curve analysis indicated that the OS rate of patients with CM with low-risk scores was remarkably longer than those patients with high-risk scores among the age ≤ 65, gender (male and female), N 0–1, T 2–4, and stage 2–4. However, the OS rates of patients with CM aged > 65, N 2–3, T 0–1, and stage 0–1 were similar between the low- and high-risk groups. These results demonstrate that the risk score based on ARLs can independently analyze the prognosis related to the different clinicopathological characteristics of CM.

### Functional enrichment analysis

**Figure 6:**
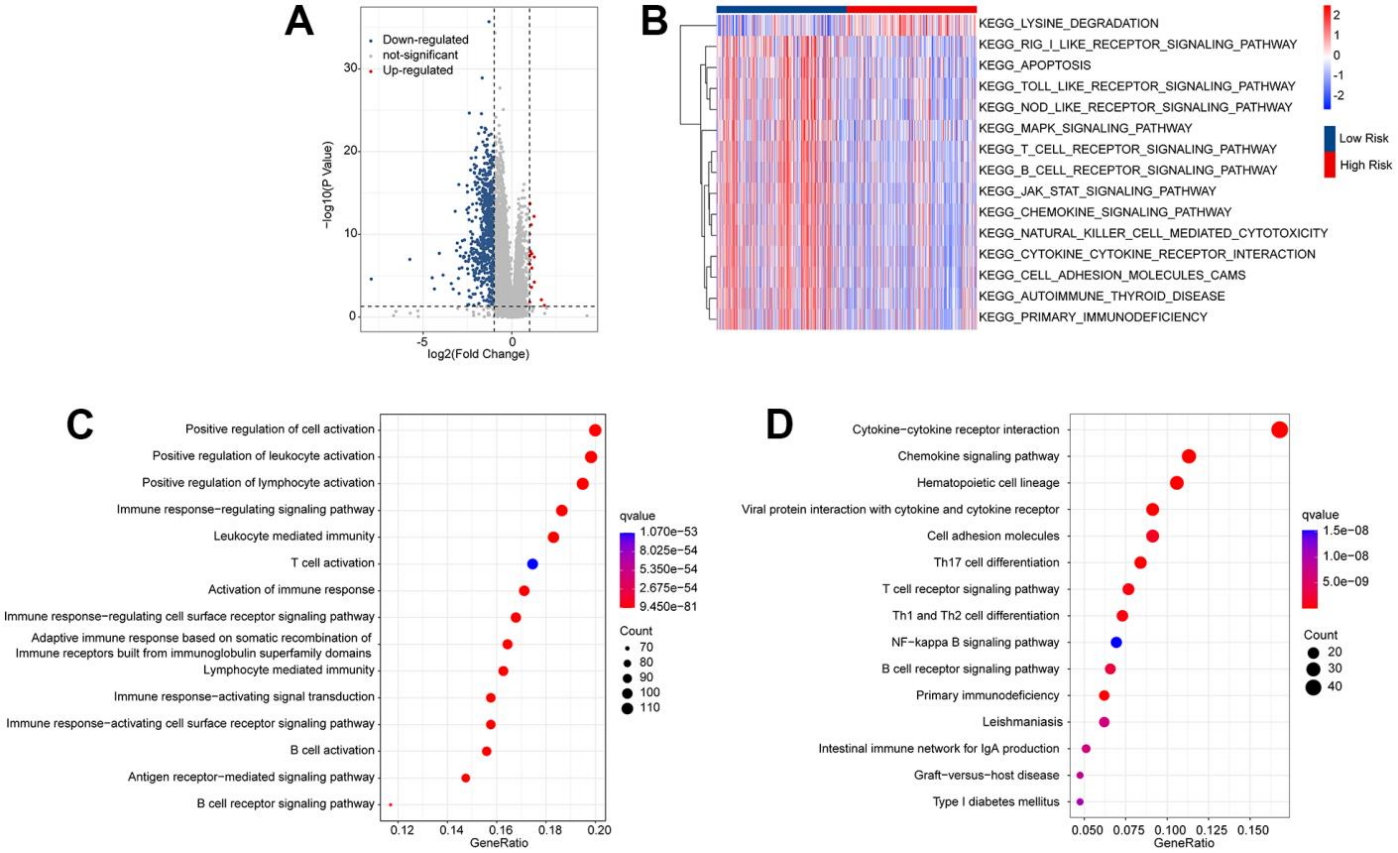
**Functional enrichment analysis of the differential expressed genes (DEGs) in the low- and high-risk group.** (**A**) Volcano diagram shows the DEGs in the low- and high-risk group with the threshold set at |Fold change| ≥ 1 and *p*-value < 0.05. (**B**) GSVA illustrates the KEGG terms of each CM patient in the low- and high-risk group. (**C**) GO enrichment analysis shows the top 15 biological processes (BP) of the DEGs. (**D**) KEGG enrichment analysis reveals the top 15 enrichment signaling approaches of the DEGs.

Enrichment analysis and GSVA were used to analyze the potential molecular mechanisms of the differentially expressed genes (DEGs) in the low- and high-risk groups. The volcano diagram illustrates the DEGs in the low- and high-risk groups; most DEGs were downregulated in the high-risk group (Figure 6A). The KEGG terms of each CM sample were analyzed using the GSVA algorithm, and the results suggested that immune-related signaling approaches were remarkably downregulated in the high-risk group (Figure 6B). GO enrichment analysis showed that the DEGs were mainly enriched in immunity-related biological processes such as positive regulation of cell activation, positive regulation of leukocyte activation, and positive regulation of lymphocyte activation (Figure 6C). KEGG enrichment analysis revealed a remarkable enrichment of DEGs in cytokine-cytokine receptor interactions and chemokine signaling pathways (Figure 6D). It is demonstrated by these results that immune-related signaling pathways may mediate the role of ARLs in CM.

### Consensus clustering analysis of ARLs associated with prognosis and immune infiltration landscape

**Figure 7:**
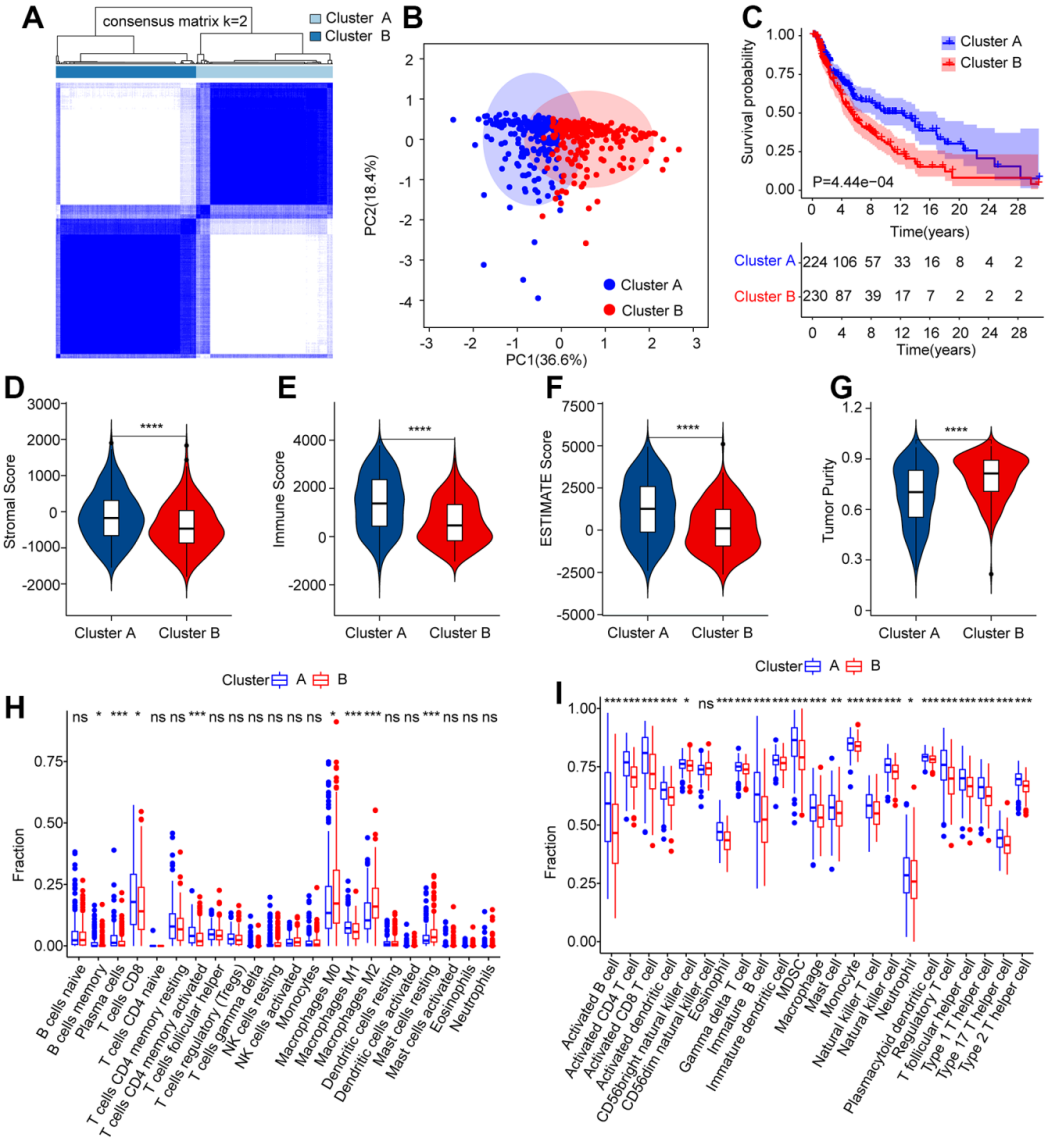
**Consensus clustering analysis of CM patients based on the prognostic ARLs.** (**A**) The consensus clustering heatmap shows the optimal classification when K = 2-9. (**B**) Principal component analysis clearly demonstrates a obvious distinction between the CM patients in Cluster A and Cluster B based on the prognostic ARLs. (**C**–**G**) The stromal, immune, ESTIMATE score and tumor purity of patients in Cluster A and Cluster B. (**H**) The fraction of 22-type immune cells of patients in Cluster A and Cluster B via CIBERSORT algorithm. (**I**) The fraction of 23-type immune cells of CM patients in Cluster A and Cluster B via the ssGSEA algorithm.

Consensus clustering analysis was conducted to cluster patients with CM into various subgroupsaccording to the prognostic ARLs. The consensus clustering heatmap indicated an optimal and stable classification with K = 2. Patients with CM were classified into two subgroups (Figure 7A). The PCA results revealed a obvious partition between Clusters A and B on the basis of prognostic ARLs (Figure 7B). The Kaplan-Meier survival curve analysis suggested that the OS rate of patients in Cluster A higher than that of patients in Cluster B (Figure 7C). Thereafter, numerious immune assessment algorithms were used to evaluate the immune infiltration landscape of patients with CM in clusters A and B. The ESTIMATE algorithm indicated that patients in Cluster A had higher stromal, immune, and ESTIMATE scores, but lower tumor purity than those in Cluster B (Figure 7D–7G). The CIBERSORT and ssGSEA algorithms were used to evaluate the immune infiltration landscape of the patients in Clusters A and B. The results of CIBERSORT illustrated that patients in Cluster A had a higher proportion of memory B cells, plasma cells, T cells CD8, T cells CD4 + memory cells, and macrophages M1, whereas the fraction of macrophages M0, macrophages M2, and resting mast cells was higher in Cluster B (Figure 7H). The ssGSEA results revealed that most immune cells were significantly higher in Cluster A than in Cluster B (Figure 7B). To sum up it is demonstrated that ARLs are linked to the prognosis and immune infiltration landscape in CM.

### Correlation analysis of the risk score and immune infiltration landscape

**Figure 8:**
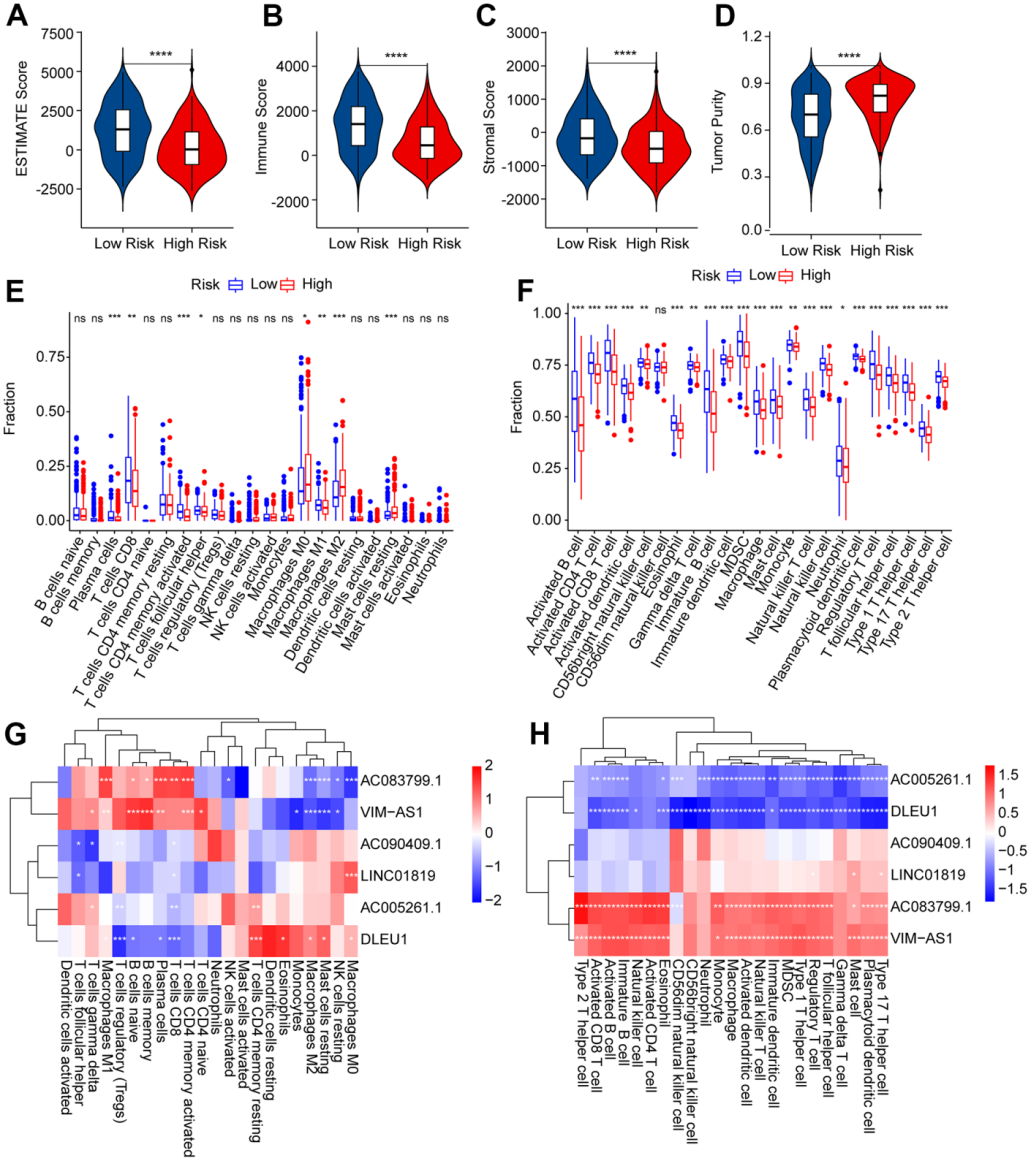
**Immune infiltration landscape of patients with CM in the low- and high-risk group.** (**A**–**D**) ESTIMATE, immune, stromal score, and tumor purity of patients with CM in the low- and high-risk group. (**E**) The fraction of 22-type immune cells of patients in the low- and high-risk group via the CIBERSORT algorithm. (**F**) The fraction of 23-type immune cells of patients in the low- and high-risk groups was analyzed by the ssGSEA algorithm. (**G**) Correlation investigation shows the relationship between the prognostic ARLs and 22-type immune cells. (**H**) Correlation investigation shows the relationship between the prognostic ARLs and 23-type immune cells.

The immune infiltration landscapes of patients in the low- and high-risk groups were further investigated. The ESTIMATE results suggested that patients with low-risk scores had higher ESTIMATE, immune, and stromal scores but lower tumor purity than those with high-risk scores (Figure 8A–8D). The CIBERSORT results indicated that the fractions of plasma cells, CD8 + T cells, CD4 + memory activated T cells, follicular helper T cells, and macrophages M1was significantly higher in the low-risk group, whereas the high-risk group had a higher proportion of M0, M2, and resting mast cells (Figure 8E). The ssGSEA results indicated that the number of most immune cells was much higher in the low-risk group than in the high-risk group (Figure 8F). Correlation analysis was performed to evaluate the connection between prognostic ARLs and immune cells, and the results reveal d a remarkable correlation between prognostic ARLs and 22-type immune cells, as calculated by the CIBERSORT algorithm (Figure 8G). Notably, the six prognostic ARLs were significantly correlated with the 23-type immune cells using the ssGSEA algorithm (Figure 8H): AC005261.1 and DLEU1 were negatively correlated with most immune cells, whereas AC083799.1 and VIM−AS1 were positively correlated with immune cells. It is demonstrated that a risk model on the basis of prognostic ARLs has close connection with the immune infiltration landscape and can show the immune status of patients with CM.

### Risk score was correlated with immunotherapy response

**Figure 9:**
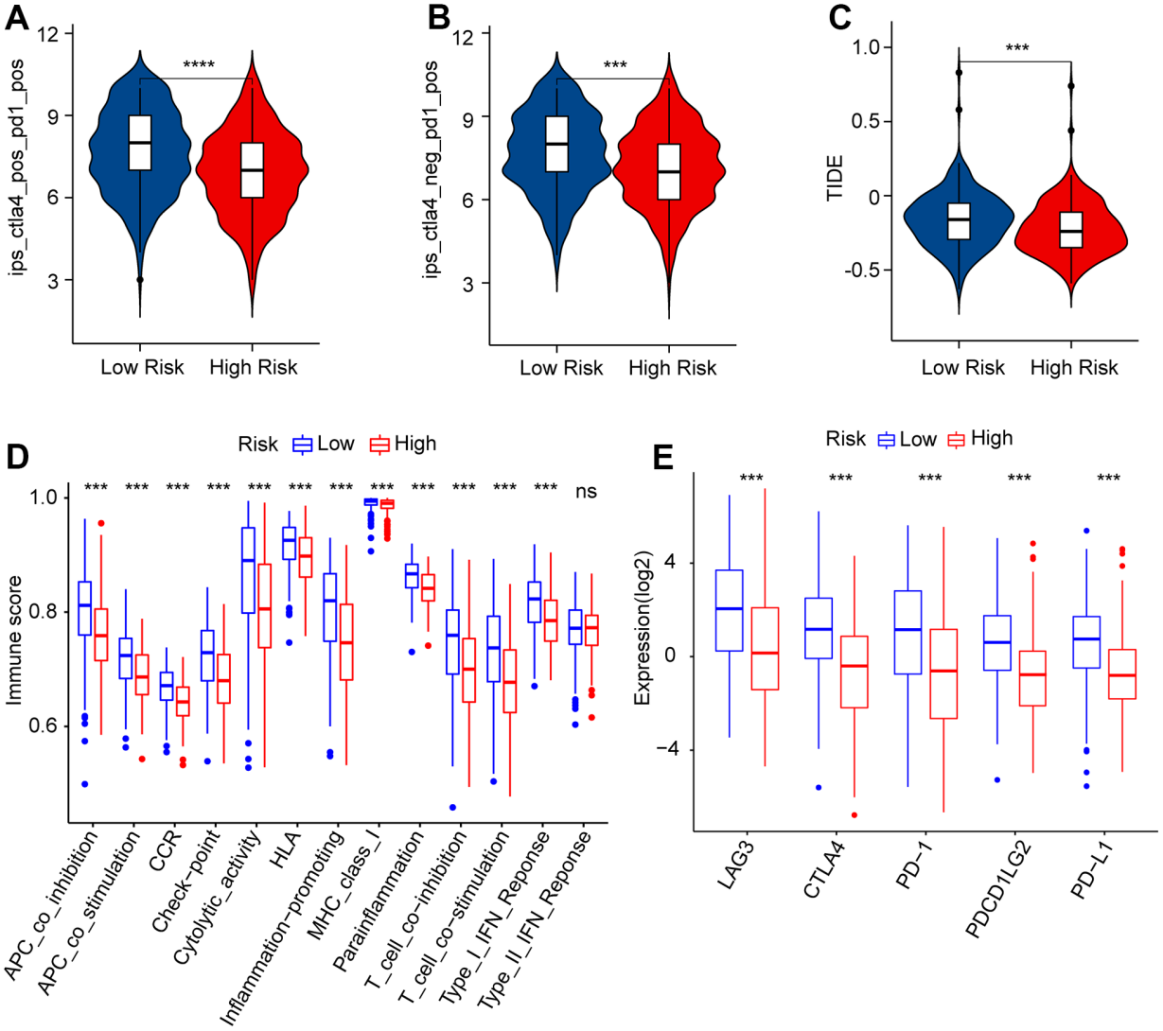
**Immunophenoscore (IPS) and immune scores of patients in the low- and high-risk group.** (**A**, **B**) IPS shows the immunotherapy reaction of CM patients in the low- and high-risk group. (**C**) TIDE score. (**D**) immune function score. (**E**) The expression of immune checkpoint inhibitors (ICI) in the low- and high-risk group. The expression is transformed by log_2_(expression + 1).

It is believed that immunotherapy is the most optimistic approach for the clinical management of CM. Given the significant differences in the immune microenvironments of patients with CM between the low- and high-risk groups, the immunotherapy responses of patients in the risk subgroups were further estimated. As shown in Figure 9A, 9B, the IPS results suggested that patients in the low-risk group showed a promising response to anti-PD-1 and anti-CTLA4/anti-PD-1. Moreover, patients with low-risk scores had higher TIDE scores than those with high-risk scores, suggesting a better immunotherapy response in CM patients with high-risk scores (Figure 9C). Immune score analysis suggested that patients in the low-risk group got higher immune functional scores than those in the high-risk group, such as those with cytolytic activity, inflammation promotion, and MHC class I (Figure 9D). The results of ICI indicated that the *LAG3*, *CTLA4*, *PD-1*, *PDCD1LG2*, and *PD−L1* expressions were significantly higher in patients with low-risk scores compared to those patients with high-risk scores (Figure 9E). These results clarify that the risk score in line with prognostic ARLs is associated with immunotherapy response, providing a fresh perspective for future precision immunotherapy for CM patients individually.

### Drug sensitivity analysis

**Figure 10:**
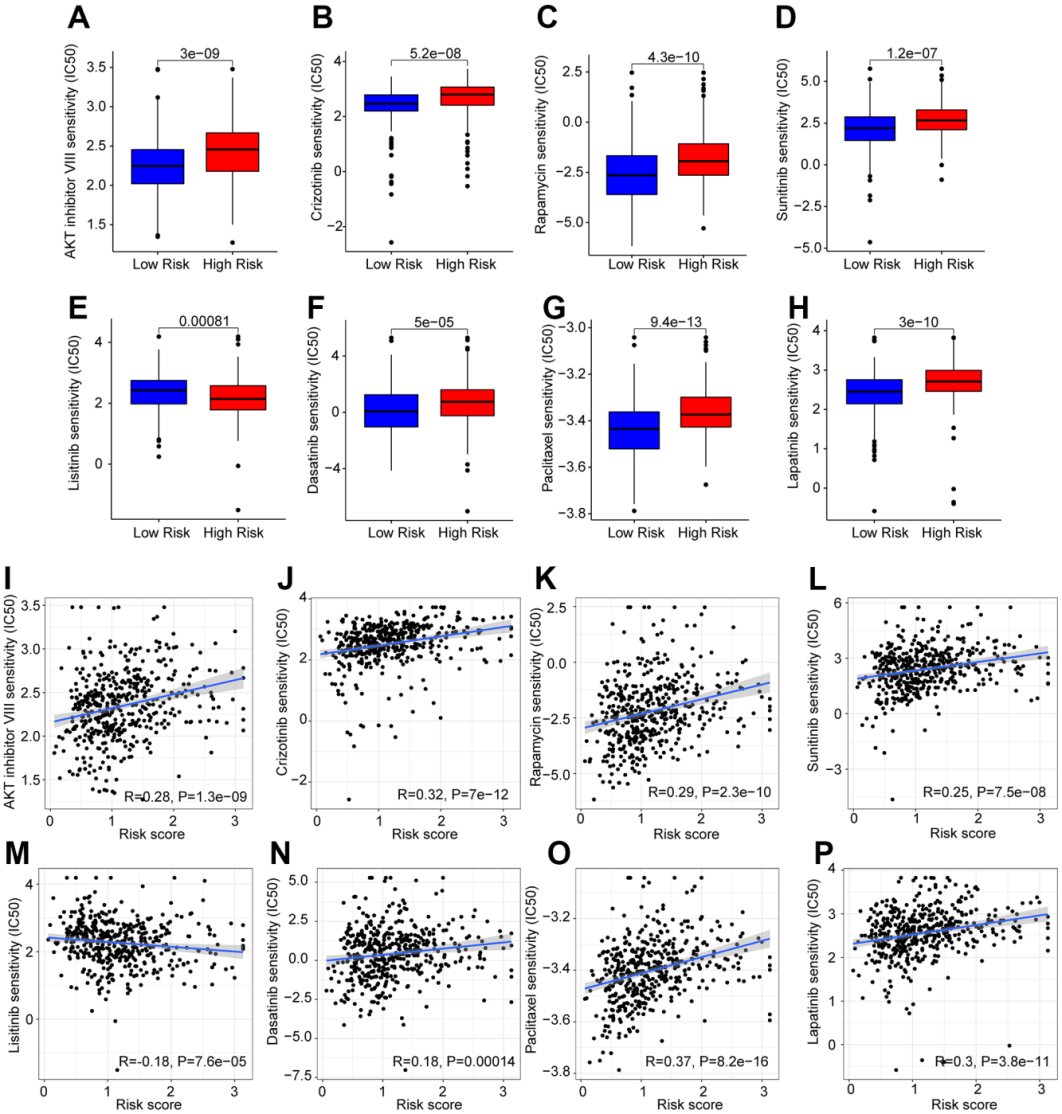
**Drug sensitivity analysis in the low- and high-risk group.** The IC50 classification reveals a remarkable distinction between patients in the low- and high-risk groups among (**A**) AKT inhibitor VIII; (**B**) Crizotinib; (**C**) Rapamycin; (**D**) Sunitinib; (**E**) Linsitinib; (**F**) Dasatinib; (**G**) Paclitaxel; (**H**) Lapatinib. (**I**–**P**) Correlation analysis of the drug sensitivity and risk score.

Targeted drug therapy is another crucial strategy in the clinical management of CM. Several potential antineoplastic drugs were identified, which had great influence in targeted therapy, and the correlation between the antineoplastic drug sensitivities and risk was further analyzed, as shown in Figure 10A–10H. The IC50 values of AKT inhibitor VIII, Crizotinib, Rapamycin, Sunitinib, Dasatinib, Paclitaxel, and Lapatinib were higher in the high-risk group, whereas the IC50 value of Linsitinib was higher in the low-risk group. Correlation analysis of risk score and IC50 value revealed that the risk score had a positive correlation with AKT inhibitor VIII, Crizotinib, Rapamycin, Sunitinib, Dasatinib, Paclitaxel, and Lapatinib, but negatively correlated with Linsitinib (Figure 10I–10P). Taken together, the results above demonstrate another response to antineoplastic drugs for CM patients in different risk subgroups, providing fresh insights into the targeted drug therapy for CM patients.

## DISCUSSION

Unresolved tumor invasion and metastasis are responsible for most cancer-related deaths [37]. CM is the most aggressive and metastatic malignant tumor and is associated with a high lethality rate [38]. Although some modern therapies, such as targeted drugs and immunotherapy, can improve the prognosis of some patients with CM, the survival rate of patients with metastatic CM is still not ideal [8]. Therefore, the inchoate detection, diagnosis, and treatment of CM should be the focus of future treatment of CM.

Anoikis is indispensable for maintaining tissue homeostasis and mainly controls cell adhesion and survival through integrin sensing and transduction of extracellular matrix signals [15]. In our study, anoikis was also strongly associated with risk in patients with CM, and patients with high-risk CM were inclined to have a lower OS. Combined with the analysis of ARLs in this study, it is further indicated that studying the role of anoikis in CM will be helpful for the clinical diagnosis and treatment of CM.

According to this study, we identified six ARLs with prognostic value by univariate Cox, Lasso, and multivariate Cox analyses, including four protective factors (AC083799.1, VIM−AS1, AC005261.1, and LINC01819) and two risk factors (DLEU1 and AC090409.1). A novel prognostic model was constructed based on these six ARLs, which had better predictive accuracy for the prognosis of CM than other clinical features. The enrichment results indicated that immune-related signaling approaches may mediate the role of ARLs in CM. The differences in the immune landscape among patients at different risks suggest that not only can prognostic models precisely predict the prognosis of CM but also reveal the immune status of CM patients. Immunotherapy and drug sensitivity analyses provided a reference for treating patients with CM. Overall, this study contributes a new model for CM that will be helpful to the early detection and diagnosis of CM and provide new insights for the individualized treatment of CM.

AC083799.1 is considered a protective factor in endometrial cancer and its expression is associated with autophagy and immunity [39]. Consistent with our results, AC083799.1 also accumulates as a protective factor in patients with CM with low-risk scores, and it is clear that there is a strong connection between anoikis and autophagy. Vimentin (VIM) is a mesenchymal marker implicated in the development of various tumors [40]. VIM-AS1 is transcribed from the VIM locus and positively regulates VIM expression [41]. Notably, studies have revealed that VIM-AS1 also interferes with tumor regulation [41]. In our experiments, the regulation of tumors by VIM-AS1 was based on a novel regulatory pathway, the anoikis pathway. LINC01819 is an lncRNA with prognostic value in the metastasis of lung tumors [40], and affected the metastasis of CM in our experiments. DLEU1 play an important role in the proliferation, migration, invasion, and inhibition of apoptosis of cancer cells [42]. The abnormally elevated DLEU1 expression in the CM may be directly related to anoikis. Our study showed that ARLs have close connection with tumorigenesis and metastasis in patients with CM.

CM is one of the most immunogenic tumors; therefore, immunotherapy has been incorporated into the treatment of CM [43]. However, CMs also acquire various inhibitory mechanisms to escape immune inspection and destruction [43, 44]. Combined with our study, although CM patients having had higher immune scores and immune cell infiltration were those who were with low scores, TIDE suggested that CM patients with low scores were inclined to have tumor immune escape, which suggested that CM patients with high-risk scores may respond better to immunotherapy. Owing to in-depth research on CM, many new therapeutic targets have been discovered, and the synergistic combination of targeted therapy and immunotherapy has gradually become the mainstream choice for CM treatment [8]. In our study, patients with CM with different risk scores showed different sensitivities to the same drug. Considering our experimental results and the current increasing availability of combined immunotherapy for CM, the feasibility of individualized treatment for patients has likewise increased, which may be beneficial for highly aggressive, highly refractory, advanced, and metastatic CM. In fact, given the important role of anoikis in early metastasis of CM, there are several ongoing CM clinical trials targeting anoikis resistance [45]. However, the unsatisfactory response rate in CM patients warrants further investigation into the causes of the differences.

Differences in drug resistance between our risk stratifications offer the possibility of contributing to differences in clinical trial outcomes. For example, the results showed differences in the sensitivity of AKT pathway inhibitors at different risk stratifications. PI3K-Akt signaling in CM is thought to contribute to anoikis resistance [46, 47]. Specific inhibition of Akt rescues anoikis resistance induced by knocking down α2, α3β1, or α5β1 integrin [48]. Activation of AKT activity by directly inhibiting AKT upstream PTEN may promote anoikis resistance [49]. Given the current poor response rate of AKT inhibitors to CM, analysis of sensitivity differences through anoikis may help in the selection of individualized treatment regimens [50].

Resistance to anoikis is a tumor metastasis hallmark, enabling tumor cells to spread through the circulatory system to distant organs [51]. Tumor cells survive anoikis through para-autocrine and paracrine mechanisms after detaching from extracellular matrix adhesion and cell-to-cell contact and regaining the ability to attach, spread, metastasize, and invade [52]. Notably, there is growing evidence that poor prognosis in malignancies is often associated with anoikis [53, 54]. The glutamate-lysing enzyme glutamate dehydrogenase 1 (GDH1) confers anoikis resistance to lung cancer cells by enhancing the binding of substrates to AMPK, which makes lung cancer prone to metastasis [54]. Anoikis also plays an important role in high-grade serous ovarian carcinoma (HGSOC), and the inhibition of anchorage-independent proliferation and the enhancement of anoikis-dependent apoptosis may be helpful in the treatment of HGSOC [55]. Anoikis promotes cancer stem cell properties of prostate cancer, therefore improving the survival of circulating tumor cells and promoting early metastasis [56]. In breast cancer, it promotes anoikis resistance in tumor cells by maintaining redox homeostasis and inhibiting JNK1 activation [57]. Combined with the ARL-based risk model with significant prognostic significance in this study, further studies on the role of ARLs in tumors will help obtain a better clinical prognosis in CM.

In conclusion, we established and validated a prognostic signature on the basis of six prognosis-related ARLs that could be used to predict OS and respond to immune status in patients with CM. More importantly, they may play a key role in treating CM. This study provides new perception into the precise diagnosis and treatment of CM.
